# FIFTEENTH PLENARY MEETING OF ISO/TC 108 ON MECHANICAL VIBRATION AND SHOCK

**DOI:** 10.6028/jres.092.028

**Published:** 1987-08-01

**Authors:** M. R. Serbyn

**Affiliations:** Automated Production Technology Division, Center for Manufacturing Engineering, National Bureau of Standards, Gaithersburg, MD 20899

The fifteenth plenary meeting of the International Standards Organization (ISO)/Technical Committee (TC) 108 on Mechanical Vibration and Shock was held at NBS Gaithersburg from March 30 through April 10, 1987. Approximately 130 delegates from 15 countries participated in the two weeks of meetings of the technical committee its four subcommittees, and nearly all of its 27 working groups.

The American National Standards Institute (ANSI), the U.S. member body, has held the secretariat for this committee since the latter was established in 1963. Presently, 17 countries are participating and 26 others are observer members. The committee maintains liaison with other TC’s of ISO and of the International Electrotechnical Commission (IEC. The chairman for the current term is Robert G. Bartheld of Siemens Energy & Automation; the secretary is Avril Brenig, standards manager of the Acoustical Society of America, which organization has administered the TC 108 Secretariat on behalf of ANSI.

Opening the plenary session, the director of NBS, Ernest Ambler, reviewed the contributions to standardization of ISO in general and of TC 108 in particular; he also stressed the communality of interests between NBS and the voluntary standards community. The working meetings that followed addressed standardization in such diverse areas as balancing machines, vibration control on vehicles and structures, calibration of vibration and shock measuring instruments, and evaluation of human exposure to mechanical shock and vibration. Those standards ranged from some very specific problems, such as “rotor-shaft-key convention,” to rather general ones, like "mechanical transmissibility of the human body in the z-direction.”

In response to the needs of manufacturers and users, new work items were established, for example, classification of environmental vibration conditions, of concern to users and makers of sensitive equipment, including computers. Several new working groups were created, while others, having completed their tasks, were dissolved. The importance of cooperation and liaison between WG’s and TC’s was emphasized. A new joint working group, consisting of experts from TC 108 and TC 70 on Internal Combustion Engines, will be called into existence to standardize the measurement and evaluation of vibration of reciprocating machines. Also, the collaboration with IEC/SC50A in the development of procedures to assess the shock resistance of mechanical systems will be continued. In all, 30 resolutions pertaining to confirmation or upgrading of standards, establishment of new work items, and creation or dissolution of working groups were adopted at the closing plenary meeting of TC 108.

In the closing meeting, Rasa Rajeswaran, representative of the ISO Central Secretariat, expressed appreciation to NBS and to ANSI on behalf of all the delegates. The meeting was closed by William Rockwell, vice president of ANSI. The next plenary meeting is to be held in Shanghai, China, in September 1988.

